# Adolf Lorenz und sein Mentor Eduard Albert

**DOI:** 10.1007/s10354-020-00752-3

**Published:** 2020-05-20

**Authors:** Gerold Holzer, Helena Kokešová

**Affiliations:** 1 Universitätsklinik für Orthopädie und Traumatologie, Allgemeines Krankenhaus, Medizinische Universität Wien, Währinger Gürtel 18–20, 1090 Wien, Österreich; 2 Masaryk-Institut und Archiv der Akademie der Wissenschaften der Tschechischen Republik (Masarykův ústav a Archiv Akademie věd České republiky), Gabčíkova 10, 182 00 Prag 8, Tschechien

**Keywords:** Wiener Medizinische Schule, Orthopädie, „Unblutige“ Operation, Kunst, Memoiren, Vienna Medical School, Orthopaedics, „Unbloddy” surgery, Art, Memoirs

## Abstract

Am Ende des 19. Jahrhunderts ermöglichte die Reichshauptstadt der k.k. Donaumonarchie, Wien, die Begegnung zweier bedeutender Persönlichkeiten der Medizingeschichte: des Chirurgen Eduard Albert (1841–1900) und dessen Schüler Adolf Lorenz (1854–1946). Beide Männer wiesen vergleichbare Züge auf: Sie stammten aus bescheidenen Verhältnissen, waren außergewöhnlich begabt und sehnten sich nach einer Karriere in der medizinischen Königsdisziplin – der Chirurgie. Beide erreichten in ihren Disziplinen die höchsten Ziele, aber das Spektrum ihrer Interessen war sehr viel breiter angelegt. Über das Leben beider und ihre Kontakte zueinander soll hier berichtet werden. Dabei konnte auch das „Gedenkbuch des Hauses Nr. 528“ von Eduard Albert in Senftenberg ausgewertet werden.

## Summary

At the end of the nineteenth century, the imperial capital of the k.k. Danube monarchy, Vienna, enabled the meeting of two important figures in medical history: the surgeon Eduard Albert (1841–1900) and his student Adolf Lorenz (1854–1946). Both men had comparable traits: they came from humble backgrounds, were exceptionally talented and longed for a career in the supreme medical discipline—surgery. Both achieved the highest goals in their disciplines but the spectrum of their interests was much broader. The life of both and their contacts to each other are reported here. In this respect the *Memorial Book of House No. 528* by Eduard Albert in Senftenberg was also evaluated.

**Figure 1:**
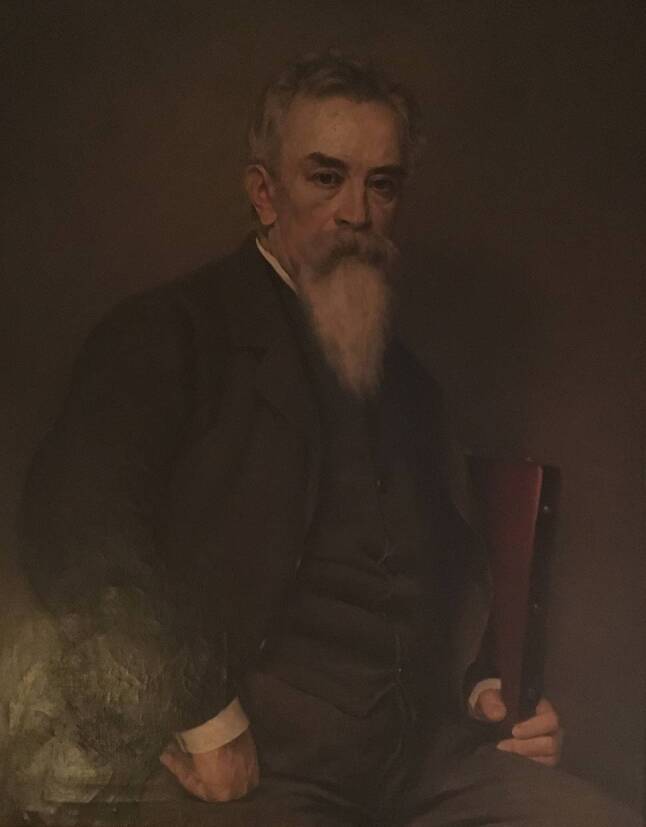

Im ausgehenden 19. Jahrhunderts gehörte Eduard Albert zu den international renommiertesten Ärzten Österreich-Ungarns (Abb. 1). Albert wurde 1841 am Fuße des Adlergebirges in Senftenberg (Žamberk)^1^ geboren. Seine Karriere nahm einen recht schnellen Verlauf. Nach dem Doktorat in Chirurgie 1869 habilitierte er 1872 und wurde schon im Jahr darauf Ordinarius der chirurgischen Universitätsklinik in Innsbruck. Im Jahr 1881 ernannte ihn Kaiser Franz Joseph I. zum Vorstand der I. chirurgischen Universitätsklinik in Wien. Damit begann sein zweites, fast 20 Jahre dauerndes Wirken in Wien, das auch von seiner Gegnerschaft zum anderen berühmten Repräsentanten der Wiener medizinischen Schule, Theodor Billroth, geprägt war [1].

Neben der Klinik widmete sich Albert v. a. seinen Schülern intensiv, von denen viele bekannte Ärzte wurden. Einer davon Adolf Lorenz konstatierte:

Seinen engeren Schülern, welchen er bisher Freund gewesen war, wurde er zum Vater [2, S. 135].

Albert selbst zählte Adolf Lorenz zu seinem Lieblingsschüler, auf den er sehr stolz war [3, S. 18–19].

Die Eheschließung Alberts mit seiner Gattin Marie (1845–1924) fand heimlich statt, da Assistenten nach den damaligen Gegebenheiten nicht heiraten durften. Aus der Ehe gingen 2 Kinder hervor, Sohn Georg (1869–1943) und Tochter Olga. Letztgenannte starb mit zweieinhalb Jahren an Tuberkulose. Der Vater setzte große Hoffnungen in seinen einzigen Sohn und wünschte sich von ihm ebenfalls eine Karriere als Arzt. Nach schweren innerfamiliären Auseinandersetzungen entsprach er Georgs Wunsch nach Aufgabe des Medizinstudiums.^2^

Alberts medizinisch-wissenschaftliche Interessens- und Forschungsschwerpunkte waren vielfältig. Neben anderen Gebieten der damals noch ungeteilten Chirurgie interessierte er sich besonders für die Orthopädie, eine Teildisziplin, die „von Billroth sehr abfällig beurteilt wurde, und leistete wichtige Beiträge hierzu“ [4, S. 126–127]. Seine besonderen Kenntnisse in Mathematik und darstellender Geometrie befähigten ihn, sich zahlreichen Fragen der Gelenkmechanik und Knochenarchitektur zu widmen [1, S. 9–18, 259–278, 5, S. 137–138].

Alberts publizierte Studien zur Mechanik des Hüft- und Kniegelenkes, zur Skoliose, zur Coxa vara, zu „Kniegelenksverkrümmungen“ (Genu valgum und Genu varum), aber auch zur Architektur einzelner Knochen und Knochenabschnitte [6]. Dabei sah sich „Albert dazu veranlasst, die Lehren Wolff’s [7] in vielen Punkten richtig zu stellen, [und] sie in anderen zu vertiefen“ [6]. Zusätzlich zu diesen eher theoretischen Arbeiten unterbreitete Albert „Operationsvorschläge“ zur Behandlung des Tibiadefektes, erdachte und führte Arthrodesen zur Behandlung von „Schlottergelenken, Paralysen und auch bei habituellen Luxationen mit Erfolg“ ein [6].

Zu den Patienten seiner Privatpraxis gehörten Mitglieder des kaiserlichen Hofes und weitere bedeutende Persönlichkeiten der damaligen Gesellschaft.

Alberts Interessen galten aber nicht nur der Medizin, sondern auch der Kunst. Er war ein genauer Kenner der tschechischen sowie internationalen Poesie, übersetzte Gedichte und verfasste selbst auch welche. Ohne Zweifel hatte Albert eine führende Position der sog. tschechischen Lobby in Wien inne. In seiner 8 Zimmer großen Wohnung unterhielt er einen deutsch-österreichischen und tschechischen Salon. Aus Briefen lässt sich erahnen, dass die damalige Crème de la Crème der Medizin, Literatur und Politik seinen Einladungen Folge leistete [1, S. 99–131]. Darüber hinaus erwarb er sich auch Verdienste als Mäzen [8].

**Figure 2:**
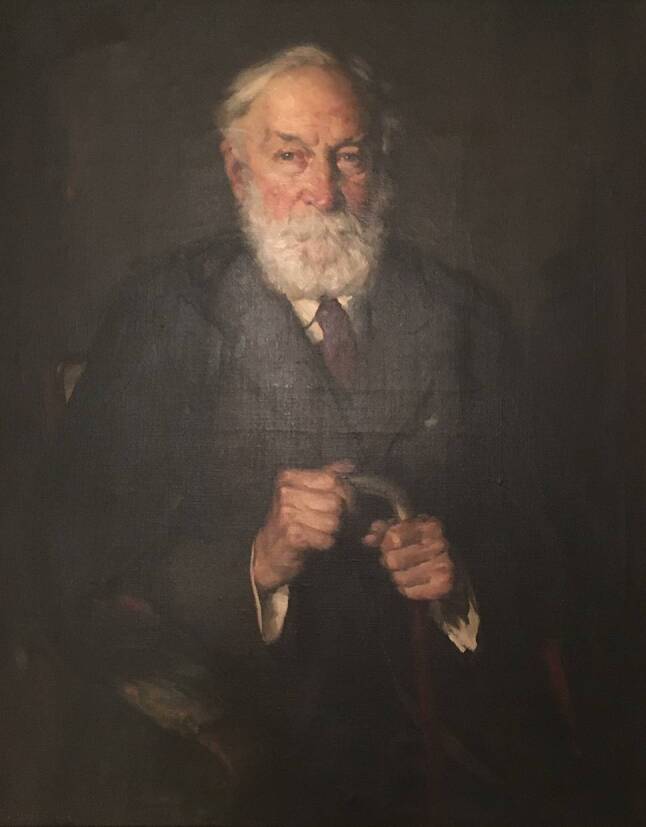

Zu Adolf Lorenz (Abb. 2) pflegte Albert enge Beziehungen, die nicht nur durch oftmaligen Kontakt, sondern vermutlich auch durch vergleichbare Lebensschicksale gespeist worden ist. Adolf Lorenz wurde 1854 in Weidenau (Vidnava) im Vorland des Altvatergebirges geboren^3^, besuchte das Gymnasium in St. Paul im Lavanttal und Klagenfurt in Kärnten [9] und studierte in Wien. Als Albert seine Tätigkeit an der Universität Wien 1881 aufnahm [10], war Lorenz Operateur, ab Oktober 1882 wurde er Alberts Assistent. Und Albert förderte Lorenz’ Karriere in vielerlei Hinsicht.

Im Oktober 1884 heiratete Lorenz Emma Lecher. Mit Emmas Vater Zacharias Konrad Lecher, selbst Arzt und später Herausgeber der Tageszeitung *Die Presse*, war auch Eduard Albert gut bekannt, da er lange Jahre in *Die Presse* publiziert hatte.

Am 02.09.1885 wurde dem Ehepaar Lorenz ein Sohn geboren. Eduard Albert war der Taufpate von Albert Lorenz, der Alberts Familiennamen als Vornamen erhielt [12, S. 20].

**Figure 3:**
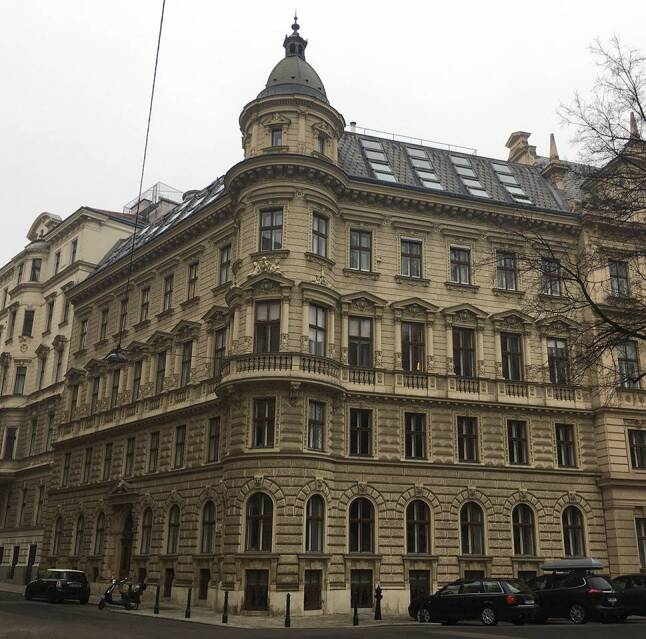

Die enge Verbindung wurde auch durch die Tatsache beeinflusst, dass Albert seit seiner Rückkehr aus Innsbruck im Jahr 1881 im Haus an der Ecke Maximilianplatz (heute Rooseveltplatz) und Frankgasse wohnte (Abb. 3). Dorthin zog später auch die Familie Lorenz. So wohnten beide Familien für einige Jahre in demselben Haus (1886–1892): Albert im ersten Stock und Lorenz im Hochparterre.^4^ Diese enge Nachbarschaft beschreibt der Sohn von Lorenz in seiner Autobiografie:

Der Herr Hofrat lebte einen Stock höher, im gleichen Haus wie wir, in der Frankgasse. Fast täglich kam er nach dem Abendessen auf ein Krügel Pilsner zu uns herunter [12, S. 20].

Da bei Lorenz durch den Gebrauch antiseptischer Desinfektionsmittel beim Operieren Karbolekzeme an den Händen Mitte der 1880er-Jahre auftraten, wurde ihm die Ausübung der klassischen Chirurgie unmöglich gemacht. Da war es Eduard Albert, der ihn auf das damals noch unbebaute Feld der Orthopädie verwies und Lorenz damit eine neue Tätigkeit ermöglichte [4, S. 130–134, 12, S. 20].

Im Kapitel mit dem bezeichnenden Titel „Durch Unglück zur ‚trockenen‘ Chirurgie“ in Lorenz’ Autobiografie erinnerte er sich mit Dankbarkeit an seinen Lehrer Albert und dessen Ratschlag:

Lieber Freund, wenn’s mit der nassen Chirurgie nicht geht, dann probieren Sie’s halt mit der trockenen [4, S. 133].

Dank Alberts Fürsprache erhielt Lorenz ein Reisestipendium und besuchte alle großen medizinischen Zentren Europas. Nach der Rückkehr übernahm er auf Professor Alberts Einladung die Behandlung der orthopädischen Fälle in seiner [Albert’s] Klinik.

Um mich [finanziell] über Wasser zu halten, verwendete er mich als Narkositarius in seiner Privatpraxis [4, S. 133–134].

Albert beriet Lorenz bei der Wahl des Habilitationsthemas und empfahl ihm, sich mit einer Arbeit aus Pathologie und Therapie zu habilitieren [4, S. 137]. Im August 1884 wurde Lorenz zum Privatdozenten an der Medizinischen Fakultät ernannt. Ende 1889 erfolgte auf Antrag der Professoren Billroth und Albert die Ernennung zum außerordentlichen Professor der Chirurgie.

Lorenz hatte also von Gedanken und Vorarbeiten Alberts profitiert, aber durch sein eigenes Tun das Fach Orthopädie etabliert. Vor allem durch „seine unblutige Operation“ (Reposition und Fixation) der kongenitalen Hüftgelenkluxation erlangte er Weltruhm [13].

Wie eigenständig seine Entwicklungen waren, lässt sich (aufgrund der heute der Öffentlichkeit zugänglichen Quellen) nicht mehr genau einschätzen. Höhepunkt seiner Karriere sollte die ab 1902 in Chicago erfolgte Therapie von Lolita Armour, der Tochter von Frau und Herrn Jonathan Ogden Armour, damals eine der reichsten Familien der Welt, sein [4, S. 283–284, 405].

Bei der Suche nach authentischen Dokumenten über Alberts Verhältnis zu Lorenz ist die wissenschaftliche Öffentlichkeit auf Quellen tschechischer Provenienz angewiesen, denn Alberts in Österreich aufbewahrtes Schrifttum ist von fragmentarischem Charakter [1, S. 18–20]. Bisher konnte nur ein einziger undatierter Brief von E. Albert an A. Lorenz mit der Bitte um eine Diagnose gefunden werden.^5^ Möglicherweise ließe sich manches Schrifttum am Privatsitz der Familie Lorenz in Altenberg finden, der jedoch nicht zugänglich ist. Dass sich keine Korrespondenz erhalten hat, könnte aber auch daran liegen, dass Albert und Lorenz enge Kollegen und Nachbarn waren und sich bei vermutlich zahlreichen Gesprächen persönlich austauschen konnten.

Als zentrale Quelle zum Verhältnis der beiden zueinander liegt nur das als „Pamětnice domu č. 528“ („Gedenkbuch des Hauses Nr. 528“) bezeichnete Besucherbuch von Alberts Villa in Senftenberg vor. Im Gedenkbuch unterschrieben nicht nur Alberts zahlreiche interessante und berühmte Gäste. Albert selbst trug dort seine Aufenthalte in Senftenberg und die Ausflüge in die Umgebung ein. In einigen Fällen beschrieb er detailliert seine Auslandsreisen, besonders die Reise nach London und Paris im Sommer 1900. Er verfasste also eine Art Chronik der wichtigen Ereignisse, sodass das Gedenkbuch für Albert vielfach die Funktion eines Tagebuchs erfüllte.^6^ Die Informationen sind leider aber sehr lückenhaft.

**Figure 4:**
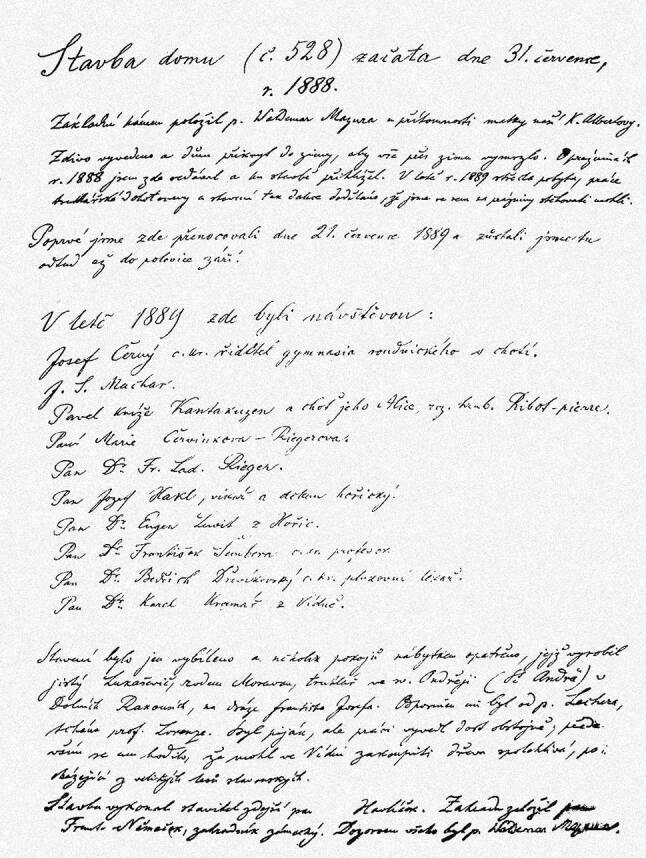

In diesem Gedenkbuch wird an einigen Stellen auch Adolf Lorenz erwähnt. Der erste Hinweis vom Sommer 1889 handelt von Bau und Ausstattung der neuen Villa in Senftenberg; an dieser beteiligte sich „ein gewisser Lukaševič, aus Mähren stammend, Tischler in St. Andrä an der Franz-Josephs-Bahn, in Niederösterreich. Empfohlen wurde er mir von Hrn. Lecher, dem Schwiegervater von Prof. Lorenz“ (Abb. 4).^7^ Dieser Eintrag zeigt, dass Albert und Lorenz nicht nur über medizinische Fachthemen sprachen, sondern sich auch mit rein praktischen Dingen beschäftigten.

Der nächste Eintrag belegt Alberts kollegialen, ja freundschaftlichen Umgang. Er führt an, während seines Ferienaufenthalts im Juli 1890 gleich von 3 Ärztekollegen besucht worden zu sein: von Rudolf Frank, Assistent der chirurgischen Klinik in Wien, Professor Adolf Lorenz aus Wien und Professor Arnold Spina aus Prag.

**Figure 5:**
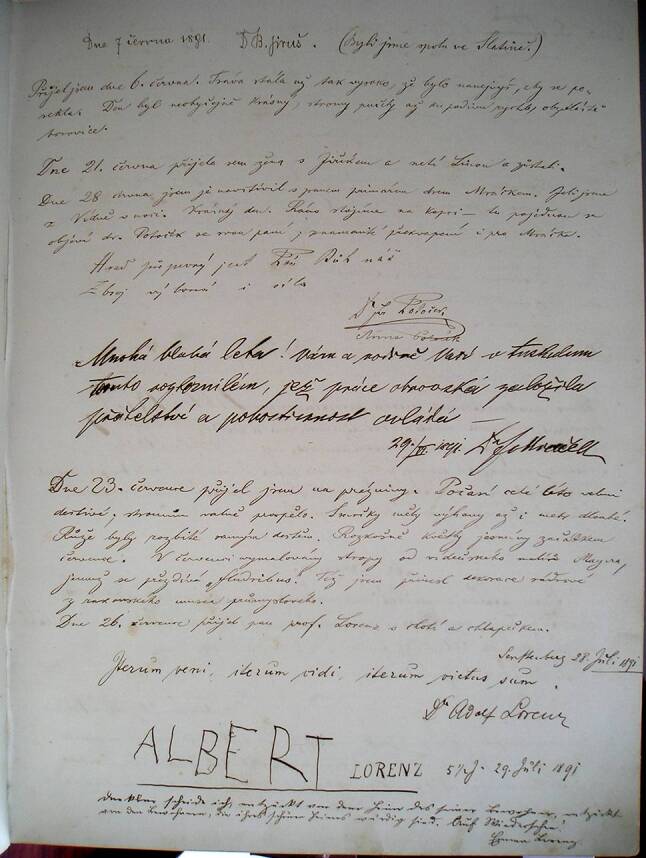

Rein privaten und freundschaftlichen Charakter dürfte wohl Lorenz’ nächster Besuch im Folgejahr gehabt haben, als er mit seiner Gattin Emma und dem 5‑jährigen Sohn Albert – dem Patensohn Eduard Alberts – nach Senftenberg reiste. Am 26.07.1891 vermerkte Albert (Abb. 5):

Es kam Herr Prof. Lorenz mit Gemahlin und jungem Sohn.

Alle, auch der kleine Albert, trugen sich in das Gedenkbuch ein.^8^

Aus weiteren Eintragungen wird ersichtlich, dass diese Besuche nicht einseitig verliefen. So verbrachte Albert auch freie Tage in der Lorenz-Villa in Altenberg. Ende September 1892 vermerkte er, dass

Lorenz mitteilt, dass die Praxis in Wien tot ist, denn die Wiener sind auf dem Land und Fremde, die sich fürchten, ob in der Zwischenzeit in Wien nicht die Cholera ausgebrochen ist, reisen nicht nach Wien.

Im Frühjahr 1895 schrieb Albert:

Einige wunderschöne Frühlingstage habe ich bei Familie Lorenz in Altenberg verbracht. Ich war dort in der Zeit der ersten Baumblüte, dann nach der Blüte der Bäume, als die Rosen blühten.

Ein weiterer Eintrag von 1895:

Im Frühjahr und im Sommer war ich häufig in Altenberg, entweder bei Familie Lorenz oder bei Familie Pflaum.^9^

Ende August 1895 erfuhr Albert aus einem Brief, dass sich Lorenz bei einem Sturz vom Pferd das Schlüsselbein gebrochen hatte.

Beide Familien begegneten sich auch bei Urlaubsreisen – so reiste Albert beispielsweise im März 1896 mit seiner Gemahlin nach Grado, wo sich auch die Ehepaare Lorenz und Hofmann aufhielten.^10^

**Figure 6:**
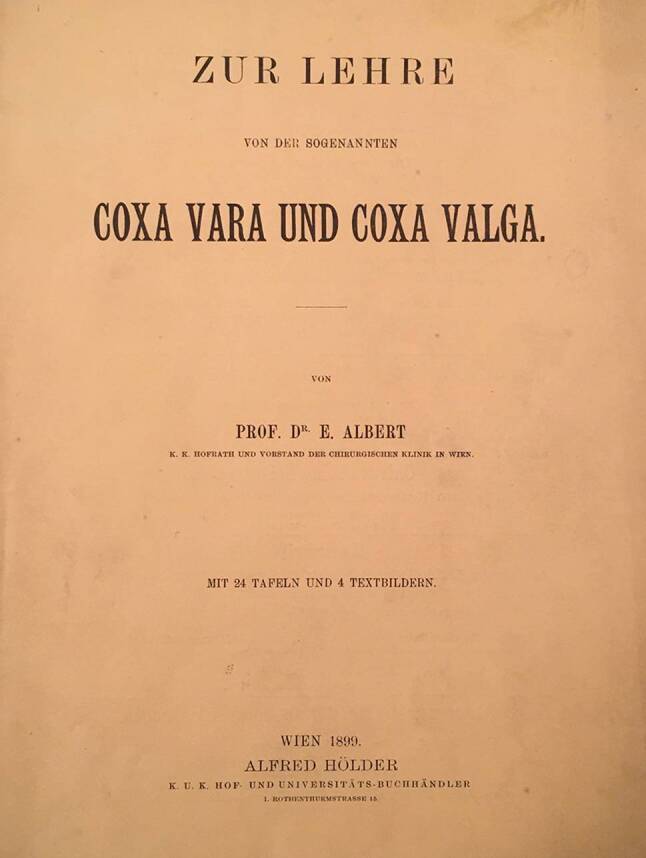

Im Gedenkbuch steht auch, dass 1899 Albert seinem Kollegen Lorenz, damals bereits Regierungsrat, die Schrift „Über die sogenannte Coxa vara und Coxa valga“ widmete (Abb. 6 und 7). Dies geschah im Gegenzug zur Dedikation der umfangreichen Lorenz-Schrift über die angeborene Hüftluxation [13].

**Figure 7:**
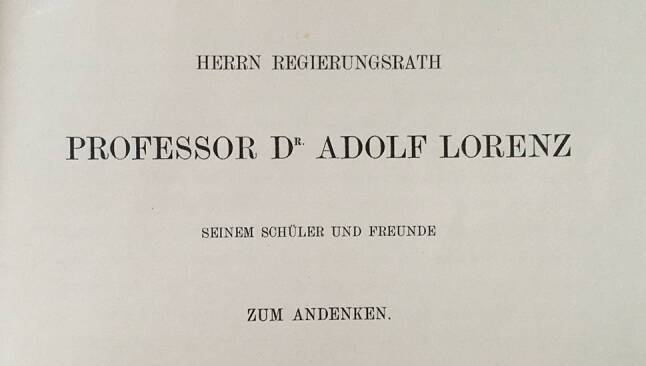

Den letzten Adolf Lorenz betreffenden Eintrag verfasste Albert nach dem 20.08.1900, als er von der Reise nach London und Paris zurückkehrte. Auf der Rückreise hätte man sich in Genf mit Familie Lorenz treffen sollen, diese war aber in der Zwischenzeit bereits nach Chamonix weitergereist.

Das Gedenkbuch schließt mit einem Eintrag von Alberts letztem Gast, Minister Antonín Rezek, der den plötzlichen Tod seines Gastgebers (in der Nacht vom 25. auf den 26.09.1900 in Alberts Heimatstadt Senftenberg) festhält.

Alberts Begräbnis am 30.09.1900 war für Senftenberg ein außergewöhnliches Großereignis. Zahlreiche bedeutende Persönlichkeiten aus Politik, Wissenschaft und Kultur sowohl von tschechischer wie von österreichischer Seite nahmen teil.^11^

Im Gedenkbuch folgen nur noch die Unterschriften der Trauergäste, die an der Beerdigung teilnahmen. Lorenz’ Unterschrift fehlt, obwohl er zum Begräbnis gekommen war und am Grab im Namen der ehemaligen Schüler gesprochen hatte.

Adolf Lorenz gedachte seines Lehrers und Förderers in einem bald nach Alberts Tod veröffentlichten Nekrolog und 3 Jahre später in einem längeren Artikel. In dem einseitigen Nekrolog, der auf der Titelseite der Wiener medicinischen Wochenschrift bereits am 29.09.1900 erschien, präsentierte er alle wichtigen Informationen in komprimierter Form [15]. Lorenz verfasste auch eine Albert-Biografie für das *Biographisches Jahrbuch und Deutscher Nekrolog* [2].

Im Jahr 1930 erschien die Publikation „Führende Chirurgen in Selbstdarstellungen“. Unter den 6 angesprochenen Medizinern befand sich auch Adolf Lorenz. In seiner Autobiografie erinnerte er sich mit Dankbarkeit an seinen Lehrer Eduard Albert und dessen Ratschlag:

Sind’s g’scheit, wenn’s mit der nassen Chirurgie nicht geht, so versuchen’s es halt mit der trockenen! So wurde ich gegen Willen und Neigung Orthopäde – und hinfort mein eigener Lehrer [16, S. 97].

Im gleichen Geist formulierte er auch in dem Artikel, den er 1936 zum Jubiläum des Allgemeinen Krankenhauses in Wien verfasste [17]. Diese Artikel dienten u. a. als Basis für die einschlägigen Passagen in Lorenz’ Erinnerungen „Ich durfte helfen“.

Eduard Albert, der in der Familie Lorenz nur als „der Herr Hofrat“ figurierte und grenzenlose Verehrung genoss, „blieb bis an sein gnädiges plötzliches Lebensende der treue Freund und Beschützer der Familie Adolf Lorenz.“ „[...] der Herr Hofrat war der Schutzpatron unserer Familie“, sagte Albert Lorenz. Er widmete Eduard Albert in seinem Erinnerungsbuch ein eigenes Kapitel, das er bezeichnenderweise „Der Herr Hofrat“ nannte [12, S. 19–27].

Mein Vater setzte seinem verehrten Lehrer im Park in Altenberg eine große steinerne Urne, die ein trauernder weiblicher Genius umfängt. Auf der Urne ist die Inschrift eingemeißelt: Eduard Albert [12, S. 24].

Daneben wuchs eine Trauerweide empor, die im Frühjahr 1944 einem Gewitter zum Opfer fiel. Die Urne soll sich noch heute im Park befinden.

Mit Eduard Alberts Lehrbüchern wuchsen mehrere Medizinergenerationen auf. In den Lehrbüchern der Chirurgie findet sich bis heute der Begriff der Albert-Naht (manchmal auch Albert-Lembert-Naht genannt). Eine Reihe seiner Facharbeiten wurde aus dem Deutschen in andere Sprache übersetzt und in immer neuen Ausgaben veröffentlicht. Die „Diagnostik der Chirurgischen Krankheiten“ erschien zuletzt 2010; das vierbändige „Lehrbuch der Chirurgie und Operationslehre“ wurde in den Jahren 2010 bis 2015 neu veröffentlicht.^12^

Beide Männer wurden postum ausgezeichnet. Alberts sterbliche Überreste wurden am 29.11.1901 von Senftenberg nach Wien überführt und auf dem Zentralfriedhof in einem von der Gemeinde Wien zur Verfügung gestellten Ehrengrab beigesetzt. Damit befand sich Albert in unmittelbarer Nähe zu seinen Freunden Eduard Hofmann und Emanuel Kusý, auch zu seinem ehemaligen Rivalen Theodor Billroth. In Prag wurde ein ganzes Stadtviertel – Albertov – nach Eduard Albert benannt.^13^ In seiner Geburtsstadt Senftenberg sind ein Platz und eine Straße nach ihm benannt, seine Namen tragen die Heilanstalt Albertinum in Senftenberg sowie Straßen in Hradec Králové (Königgrätz), Olomouc (Olmütz) und Kroměříž (Kremsier).

Adolf Lorenz starb in Altenberg und wurde in einem Ehrengrab auf dem Friedhof von St. Andrä-Wördern bestattet. In Altenberg ist die Gasse nach ihm benannt, auch in Wien gibt es seit 1959 eine Adolf-Lorenz-Gasse in Hietzing, 13. Bezirk.

## Schluss

Zwischen Eduard Albert und Adolf Lorenz bestanden enge Verbindungen, nicht nur beruflich, sondern auch familiär. Ein Grund dafür dürfte vielleicht gewesen sein, dass Eduard Albert in Adolf Lorenz und dessen Karriere wahrscheinlich die Erfüllung jener Träume sah, die er für seinen eigenen Sohn gehegt hatte. Dagegen dürfte Adolf Lorenz als erfolgreichster und bekanntester Schüler Alberts und Begründer einer neuen Disziplin dessen Erwartungen sicherlich gänzlich erfüllt haben.

## Footnotes

1 Die Stadt Senftenberg (Žamberk) gehörte zum Königreich Böhmen, ab 1918 Teil der neu gegründeten Tschechoslowakei.

2 Georg wechselte an die Wiener Philosophische Fakultät, wo er 1895 sein Studium abschloss. Die Möglichkeit Hochschullehrer zu werden, nutzte Georg nicht, obwohl seine Doktorarbeit hochgeschätzt und gedruckt wurde. Er nahm keine feste Stelle an, sondern widmete sich als Privatgelehrter seinen Forschungen zu Immanuel Kant sowie philologischen und philosophischen Studien. Außerdem war er literarisch tätig und verfasste beispielsweise Gedichte. Er war zweimal verheiratet: aus der ersten Ehe mit Maria geb. Planinschegg (1868–1919) stammten zwei Töchter und ein Sohn: Emma, verheiratete Sieberer (1904–1989), war Lehrerin und Komponistin, Edith, verheiratete Wilheim (1912–2007), war Mutter der Tochter Edith und des Sohnes Friedrich. Georgs Sohn Ernst Albert (1908–?) schloss sein Studium an der juridischen Fakultät der Universität Wien ab und ging Ende Juni 1936 nach Berlin. Im Juli 1942 wurde ihm die deutsche Staatsbürgerschaft entzogen und der Doktortitel aberkannt. Im Januar 1946 wurde er in London naturalisiert. Georges zweite Ehe mit Friederika geb. Bayer (1872–?), einer ehemaligen Volksschullehrerin, blieb kinderlos. Georg Albert starb am 19. Dezember 1943 in Wien an Magenkrebs. Wiener Stadt- und Landesarchiv, Bestand Bundespolizeidirektion Wien: Historische Meldeunterlagen und Bestand Totenbeschreibamt. The London Gazette, 8. February 1946, S. 864. https://www.thegazette.co.uk/London/issue/37461/page/1.

3 Die Stadt Weidenau befand sich im Herzogtum Schlesien (Oberschlesien). Das Herzogtum Ober- und Niederschlesien war ein inkorporierter Teil der Länder der Böhmischen Krone und damit der Habsburgermonarchie. Im Jahr 1918 fiel ein Großteil sgn. Österreichisch-Schlesiens der Tschechoslowakei zu.

4 Eduard Albert wohnte zunächst an der Adresse Maximilianplatz 7/Frankgasse 2; später – im August 1899 – zog er 2 Häuser weiter in die Nummer 10. Siehe Wiener Stadt- und Landesarchiv, Bestand Bundespolizeidirektion Wien: Historische Meldeunterlagen, Sign. 2. 5. 1. 4, vom 28.09.1899. Siehe auch [11].

5 Geschrieben ist der Brief auf einem Formular mit dem vorgedruckten Text: „Prof. Dr. E. Albert, k. k. Hofrath und Ober-Sanitätsrath, Vorstand der I. chirurgischen Universitäts-Klinik und des Operateur-Institutes, IX. Bez., Maximilianplatz Nr. 7/Frankgasse Nr. 2. Ordination von 3 bis 4 Uhr. (Für Arme unentgeltlich im k. k. allgemeinen Krankenhause von 10–12 Uhr.)“. Österreichische Nationalbibliothek, Sammlung von Handschriften und alten Drucken, Abteilung Handschriften, Autographen und Nachlässe, Sammlung Otto Frankfurter, Sign. Autogr. 217/1–2 Samml.: Han. Im Lorenz-Archiv der Adolf und Albert Gedenkstätte werden nur Sonderdrucke einiger Arbeiten von Eduard Albert verwahrt. Adolf und Albert Lorenz Gedenkstätte, Rathausstraße 21, Wien. http://www.medacad.org/adolf-lorenz/index.html.

6 Městské muzeum Žamberk (Stadtmuseum Senftenberg), Nachlass E. Albert, Pamětnice domu č. 528 (Gedenkbuch des Hauses Nr. 528), nicht paginiert.

7 Ibid. Die Zitate im Text sind hier aus dem Tschechischen übersetzt.

8 „Senftenberg 28. Juli 1891. Iterum veni, iterum vidi, iterum victus sum. Dr. Adolf Lorenz ALBERT Lorenz 5 ½ J. 29. Juli 1891. Dankbar scheide ich, entzückt von dem Heim das seiner Bewohner, entzückt von den Bewohnern, die ihres schönen Heimes würdig sind. Auf Wiedersehen! Emma Lorenz.“ Ibid.

9 Ibid.

10 Gemeint ist die Familie des Gerichtsmediziners Professor Eduard Hofmann (1837–1897), der aus Prag stammte und maßgeblich an Alberts Berufung an die Universität Wien beteiligt gewesen war. Über dieses Treffen schrieb Albert an den Journalisten Gustav Eim am 30.03.1896 [14, S. 198–199].

11 Státní okresní archiv Ústí nad Orlicí (Staatliches Bezirksarchiv Wildenschwert), Archivbibliothek und Bestände (Notářství Žamberk; Archiv města Žamberk; Děkanský úřad Žamberk; Obecná škola Žamberk). Městské muzeum Žamberk (Stadtmuseum Senftenberg), Nachlass E. Albert, Pamětnice domu č. 528 (Gedenkbuch des Hauses Nr. 528). Vgl. auch die zeitgenössische Presse. Einen Überblick über die Teilnehmer liefert [1, S. 249–254].

12 https://www.amazon.co.uk/Alberts-Diagnostik-Chirurgischen-Krankheiten/dp/1145034780. https://www.payot.ch/Detail/diagnostik_der_chirurgischen_krankheiten_in_zwanzig_vorlesungen_german_edition-eduard_albert-9781142865580 und https://www.amazon.de/albert-eduard-lehrbuch-chirurgie-operationslehre/s?ie=UTF8&page=1&rh=i%3Aaps%2Ck%3Aalbert%20eduard%20lehrbuch%20der%20chirurgie%20und%20operationslehre.

13 Zu Albertov (Alberthof) siehe https://cs.wikipedia.org/wiki/Albertov.
